# WPO-Net: Windowed Pose Optimization Network for Monocular Visual Odometry Estimation

**DOI:** 10.3390/s21238155

**Published:** 2021-12-06

**Authors:** Nivesh Gadipudi, Irraivan Elamvazuthi, Cheng-Kai Lu, Sivajothi Paramasivam, Steven Su

**Affiliations:** 1 Smart Assistive and Rehabilitative Technology (SMART) Research Group, Department of Electrical and Electronic Engineering, Universiti Teknologi PETRONAS, Bandar Seri Iskandar 32610, Malaysia; nivesh_18001319@utp.edu.my (N.G.); chengkai.lu@utp.edu.my (C.-K.L.); 2 School of Engineering, UOWM KDU University College, Shah Alam 40150, Malaysia; siva@kdu.edu.my; 3 School of Biomedical Engineering, University of Technology Sydney, Ultimo 2007, Australia; Steven.Su@uts.edu.au

**Keywords:** visual odometry, pose estimation, pose optimization, deep learning

## Abstract

Visual odometry is the process of estimating incremental localization of the camera in 3-dimensional space for autonomous driving. There have been new learning-based methods which do not require camera calibration and are robust to external noise. In this work, a new method that do not require camera calibration called the “windowed pose optimization network” is proposed to estimate the 6 degrees of freedom pose of a monocular camera. The architecture of the proposed network is based on supervised learning-based methods with feature encoder and pose regressor that takes multiple consecutive two grayscale image stacks at each step for training and enforces the composite pose constraints. The KITTI dataset is used to evaluate the performance of the proposed method. The proposed method yielded rotational error of 3.12 deg/100 m, and the training time is 41.32 ms, while inference time is 7.87 ms. Experiments demonstrate the competitive performance of the proposed method to other state-of-the-art related works which shows the novelty of the proposed technique.

## 1. Introduction

Autonomous vehicles, including unmanned aerial vehicles (UAV), unmanned ground vehicles (UGV), and unmanned underwater vehicles (UUV), are increasingly used to explore the different difficult and dangerous environments to minimize human interaction. In addition, mobile robots became an integral part of the present industry evolution for logistics and supply chain management. Estimating the ego-motion or continuous localization of the robot in an environment is a fundamental long-standing challenge in autonomous navigation. Traditionally, continuous localization is performed using sensors, such as global positioning systems (GPS), inertial sensors, and wheel encoders for ground robots. Traditional methods suffer from accumulated drift and GPS is constrained to only open environments. Recent studies expressed immense interest to perform the localization task using cameras due to vast information. The method of performing the continuous localization using cameras or visual-only sensors is known as visual odometry (VO). The applications of visual odometry vary widely from scene reconstruction [1], indoor localization [2], biomedical applications [3], and virtual and augmented reality [4] to self-driving vehicles [5].

VO acts as a fundamental block of a similar set of algorithms, such as visual simultaneous localization and mapping (VSLAM) and structure from motion (SfM). State-of-the-art are the earliest methods of VO algorithms and are classified into sparse methods [6,7] and dense methods [8] based on the minimization objectives. Sparse methods use the features extracted from consecutive images to estimate the motion by minimizing reprojection errors. Dense methods concentrate on individual pixels of consecutive images to reconstruct a more comprehensive scene and work on the principle of photometric consistency. Though the state-of-the-art methods are efficient in estimating the motion, these methods require a series of complex pipelines consisting of individual components addressing the multi-view geometric tasks which require hard tuning based on the environment. A slight malfunctioning of a subcomponent can result in the degradation of the entire pipeline. However, estimating visual odometry is a multi-view geometric problem and requires knowledge of the underlying 3-dimensional (3D) structure. In addition, these methods are less generalized, which means they are not intelligent to learn from the different modalities of environments.

Considering the above shortcomings of the state-of-the-art methods, researchers of the computer vision community concentrated on alternative algorithms based on the learning paradigm. Learning-based algorithms gained massive attention due to their capability of implicitly learning the hidden representations with more generalization ability. Recently, methods using deep learning revealed superior performance over traditional methods in object classification, detection, and recognition [9,10]. Earlier learning-based methods used recurrent neural networks to improve the long-term temporal dependencies that mitigate pose drift problems [11]. On the other hand, some methods used optical flow estimates extracted from images to feed the networks [12]. The resultant of either of these are larger network parameters with high computational time. Current work deals only with monocular videos and learning-based methods using left-right consistency for training are not included in the evaluation [13,14].

The main aim of this paper is to improve pose predictions derived from convolutional neural networks given a set of images stacks and ground truths using windowed optimization. This is achieved by multiple forward passes from multiple inputs and a single back-propagation based on cumulative loss. From a point, the proposed network can be viewed as multiple siamese networks that share the same parameters among the same networks. The main contributions of this paper are:

1. A new learning-based optimization method without any additional modifications to the network is proposed.
2. Proposed network is independent of optical flow preprocessing and temporal processing modules, such as recurrent neural networks. Most importantly, WPO-Net is relatively small and consists of only 0.48 million parameters.
3. Experiments are performed to emphasize the importance of data augmentation in learning-based VO methods and the effect of varying window sizes in the proposed optimization framework.
4. Comparative experiments showcase the competitive performance of the proposed method with other geometric or state-of-the-art methods, supervised and unsupervised learning-based methods.

The paper is organized as follows: Section 1.1 presents an overview of the published related works. Section 2 describes the building blocks of the method, including network architecture, windowed pose optimization technique, and loss function. Section 3 presents details of training and testing datasets, hardware, and software environments. In addition, this section also presents the evaluation of the present method on the KITTI dataset, data augmentation, and ablation tests.

### 1.1. Related Work

VO estimation is a long-standing multi-view geometry problem. Over the years, there have been several approaches that are being used to address the task of VO estimation. These algorithms can be classified into two distinctive types, namely state-of-the-art methods and learning-based methods. State-of-the-art methods are also referred to as geometric or traditional methods, alternatively.

#### 1.1.1. State-of-the-Art Methods

State-of-the-art or geometric methods are further classified into the sparse of feature-based methods and direct or dense methods. As discussed, feature-based methods work by minimizing the reprojection error between features from consecutive frames. The feature extracted can be edges, lines, or blobs. Most famous feature extraction methods are ORB [15], FAST [16], and SURF [17]. Some of the early feature-based methods, such as in Reference [7], used filtering techniques to simultaneously optimize the map points and position of the robot. The major drawback associated with filtering-based VO/VSLAM is the increase in computational cost as the map grows. This issue was addressed by keyframe-based algorithms, which use independent threads for mapping and tracking threads [4]. These keyframe-based methods use bundle adjustment as the backbone of optimizing the position and map points to reduce drifts. Down the road, these algorithms became more efficient and are highly dependent on the robustness of feature extractors. ORB-SLAM [6] and VISO2 [18] are some of the most efficient real-time feature-based VO/VSLAM algorithms. Nevertheless, feature-based algorithms suffer from textureless and noise-induced regions. On the other hand, direct methods minimize the pixelwise reprojection error from consecutive images. Direct methods can reconstruct more comprehensive 3D scenes but are computationally expensive and limit the real-time usability of these algorithms [8,19]. A combination of direct and feature-based methods are also developed to estimate the pose using the features and the regions surrounding the pixels, and these are known as semi-direct methods [20]. However, the direct method works on the principle of photometric consistency and is not designed to deal with large viewpoint changes.

#### 1.1.2. Learning-Based Methods

Learning-based methods are the most recent VO algorithms. Due to the continuous increase in the availability of graphic processing units (GPUs), benchmark datasets, such as KITTI [21], and synthetic data generation frameworks, such as CARLA [22] and TartanAir [23], there has been a shift in increased research towards learning-based algorithms. Learning-based methods are robust to unmodeled noise and environmental changes and work by learning the hidden feature representations. Learning-based methods are further classified into supervised and unsupervised based on the learning paradigms. One of the main challenges of learning-based methods is adapting to the architectures that were being used for 2D tasks, such as classification, recognition, and localization. These architectures operate by taking a single image as input, but the VO estimation requires a stack of consecutive images.

Supervised learning-based methods rely on the ground truth 6 degrees of freedom (DOF) poses to optimize the parameters. Earliest learning-based method can be dated back to 2008 [24]. Later, the VO estimation was recognized as a regression task. The invention of architectures, such as PoseNet [25], used to regress the absolute 6 DOF pose, and FlowNet [26], used for optical flow extraction between two images, provided great support for learning-based VO estimation algorithms. Supervised learning-based methods learn the hidden mapping by taking optical flow or raw images. LS-VO [27] and Flowdometry [12] learn to predict the pose by used optical flow. However, these methods involve computationally expensive preprocessing to extract the optical flow from images. Methods, such as DeepVO [11] and PCGRU [28], used recurrent neural networks to minimize the prediction errors. Another interesting development includes uncertainty quantification in the pose prediction process [29]. DeepVO estimates the covariance matrix along with pose estimation. This work is highly motivated by the fact that this uncertainty quantification can be used to adaptively weigh the translation and rotational components of the pose estimates. Reference [30] estimates the 2 DOF pose for ground vehicles by neglecting the less significant movement along the other four axis. The proposed WPO-Net inherits some architectural design philosophies, such as rectangular convolutions from Reference [30].

On the other hand, unsupervised methods work on the foundational principle of single view image synthesis. These methods operate in complex end-to-end format involving several networks to address tasks, such as depth estimation, dynamic region masking, and pose estimates. SfMLearner [31] is designed to estimate the depth and pose by neglecting unexplainable pixels. GeoNet [32] further included the dynamic object compensation to avoid the erroneous pose estimates. CM-VO [33] proposed a confidence quantification and refining the trajectory based on the confidence. Though unsupervised methods eliminate the requirement of ground truths, the performance of these methods is not on par with the supervised learning-based methods. To address the above problems in learning-based methods, a windowed optimization approach is presented in this paper. The proposed method optimizes the pose of a short window of images using the trajectory consistency constrain and is analogous to windowed bundle adjustment in traditional methods.

## 2. Methodology

This section includes the introduction to subcomponents of the proposed method. The entire framework is composed of two subcomponents, namely a feature encoder and pose regressor. The feature encoder transforms the high-level gray images into a compact global feature descriptor. The extracted feature descriptor is transformed into a 6 DOF pose estimate by the pose regressor. Further, CNN-based windowed pose optimization and loss function used for training are explained in Section 2.4 and Section 2.5, respectively.

### 2.1. Preprocessing

The original raw grayscale input images of size 1241 × 376 are resized to 640 × 192 to meet the specifications of the proposed network and to reduce the memory consumption of the GPU. A general procedure of standardizing the images about mean and variance is used to narrow down the distribution and to pace up the convergence. Two consecutive images are stacked along the channels to serve as the input to the feature encoder. A temporal skipping strategy for augmenting the data is used by selecting a consecutive random frame within an interval of 0 to 4 in the forward direction to learn more distinctive and complicated mapping.

### 2.2. Feature Encoder

VO or continuous ego-motion estimation requires consecutive image pairs. In traditional methods, this is performed by feature matching or photometric consistency across the frames of the sequence. In learning-based methods using deep learning, the hidden representations of the images are automatically extracted to estimate the 6 DOF pose. The proposed feature encoder takes in a stack of two grayscale images of size 640 × 192 at each training step. The details of the architecture of the feature encoder used for this method are presented in Table 1.

Feature encoder consists of seven layers using the rectangular kernels, except the last one. A combination of different strides and dilations are used to efficiently reduce the size of the network by extracting the features with greater receptive coverage. The last layer is a special convolutional pooling layer to downsample the dimensions of the descriptor. Batch normalization and ELU (exponential linear unit) are used for every layer to accelerate the convergence.

### 2.3. Pose Regressor

The extracted global feature descriptor from the feature encoder is transformed into a 6 DOF pose estimate by feeding into a two-layered MLP (multilayer perceptron). The first layer consists of 256 nodes with ELU activation. The output or the second layer of the pose regressor consists of 6 nodes with linear activation. The output vector represents the translations and rotations in Euler angles about each axis. The predicted values are quantitatively used to estimate the loss with the labeled ground truth.

### 2.4. Windowed Pose Optimization

**Figure 1:**
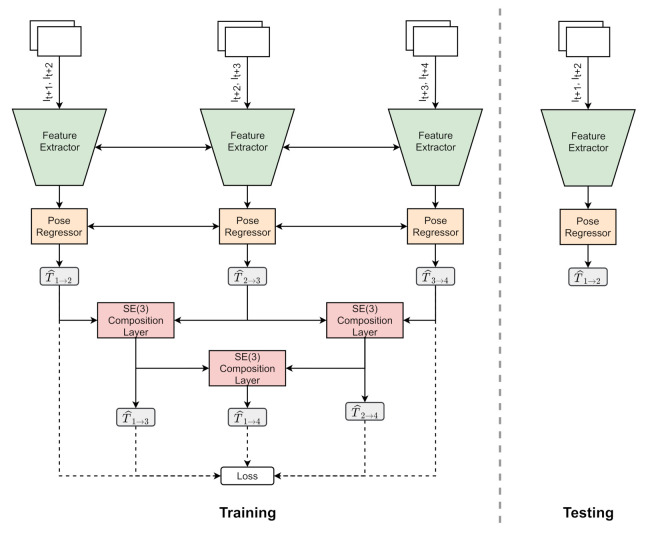
Overview of the WPO-NET. SE(3) composition layer is used to derive the composite poses from predicted poses.

Proposed approach adopts a unique strategy motivated by the benefits of windowed bundle adjustment in reducing drifts. The proposed networks use four images of the video sequence and stack them into 3 overlapping samples to feed the network. Let $\left\{I_{t},I_{t+1},I_{t+2},I_{t+3}\right\}$ be the four consecutive images stacked into $\left\{I_{t,t+1},I_{t+1,t+2},I_{t+2,t+3}\right\}$, as shown in Figure 1. First, each training iteration consists of forward propagating a triplet network using three consecutive image stacks. Second, the gradients are propagated backward by estimating the cumulative loss of predictions from triplets. A detailed explanation of the formulated loss function used for training is presented in Section 2.5. A SE(3) composition layer is used to estimate the implicit transformations $\left\{T_{t\to t+2},T_{t\to t+3},T_{t+1\to t+3}\right\}$ of unrelated stacks during training. SE(3) composition layer is used to transform the predicted 6 DOF pose estimate se(3) into SE(3) transformation matrix, and vice versa, ensuring the differentiable properties. The elements of se(3) can be mapped to SE(3) by using an exponential map and SE(3) to se(3) using the logarithmic map.

Consider $u=\left[x,y,z,\omega_{1},\omega_{2},\omega_{3}\right]\in \mathrm{se}\left(3\right)$, where (x,y,z) and $\left(\omega_{1},\omega_{2},\omega_{3}\right)$ representing the translations and Euler angles. The corresponding generators of se(3) representing the derivatives of translations and rotations about each axis can be formulated as Equation (1):

(1) $$\begin{array}{c} \begin{array}{c} G_{1}\;=\;\left(\begin{array}{cccc} 0 & 0 & 0 & 1\\ 0 & 0 & 0 & 0\\ 0 & 0 & 0 & 0\\ 0 & 0 & 0 & 0 \end{array}\right),\;G_{2}\;=\;\left(\begin{array}{cccc} 0 & 0 & 0 & 0\\ 0 & 0 & 0 & 1\\ 0 & 0 & 0 & 0\\ 0 & 0 & 0 & 0 \end{array}\right),\;G_{3}\;=\;\left(\begin{array}{cccc} 0 & 0 & 0 & 0\\ 0 & 0 & 0 & 0\\ 0 & 0 & 0 & 1\\ 0 & 0 & 0 & 0 \end{array}\right),\\ G_{4}\;=\;\left(\begin{array}{cccc} 0 & 0 & 0 & 0\\ 0 & 0 & -1 & 0\\ 0 & 1 & 0 & 0\\ 0 & 0 & 0 & 0 \end{array}\right),\;G_{5}\;=\;\left(\begin{array}{cccc} 0 & 0 & 1 & 0\\ 0 & 0 & 0 & 0\\ -1 & 0 & 0 & 0\\ 0 & 0 & 0 & 0 \end{array}\right),\;G_{6}\;=\;\left(\begin{array}{cccc} 0 & -1 & 0 & 0\\ 1 & 0 & 0 & 0\\ 0 & 0 & 0 & 0\\ 0 & 0 & 0 & 0 \end{array}\right). \end{array} \end{array}$$

For mathematical convenience, we denote translations *u* and rotations ω separately. The linear combinations of generators can written as Equation (2):

(2) $$\delta =\left(p\;\omega \right)=xG_{1}+yG_{2}+zG_{3}+\omega_{1}G_{4}+\omega_{2}G_{5}+\omega_{3}G_{6}\in \mathrm{se}\left(3\right),$$

where $G_{1},G_{2},G_{3}$ are partial derivatives of translations about X,Y,Z axis with linear combinations $p=xG_{1}+yG_{2}+zG_{3}$, respectively. $G_{4},G_{5},G_{6}$ are partial derivatives of Euler angles $\left(\omega_{1},\omega_{2},\omega_{3}\right)$ on the X,Y,Z axis with linear combinations $\omega =\omega_{1}G_{4}+\omega_{2}G_{5}+\omega_{3}G_{6}$, respectively. The linear combinations of generators representing δ=(pω)∈se(3) are transformed to SE(3) by applying the exponential mapping

(3) $$\exp\left(\delta \right)=\left(\begin{array}{cc} e^{\omega_{x}} & Vp\\ 0 & 1 \end{array}\right).$$

Using Taylor expansion, exponential map of ω and *V* can be formulated as:

(4) $$\begin{array}{c} e^{\omega_{x}}=I_{3}+\frac{\sin\theta }{\theta }\omega_{x}+\frac{1-\cos\theta }{\theta^{2}}\omega_{x}^{2},\\ V=I_{3}+\frac{1-\cos\theta }{\theta^{2}}\omega_{x}+\frac{\theta -\sin\theta }{\theta^{3}}\omega_{x}^{2}, \end{array}$$

where $\theta \;=\;\left|\omega \right|$, $\omega_{x}$ is the skew-symmetric matrix from the linear combination of rotational generators. Similarly, $T\;=\;\left(\begin{array}{cc} R & t\\ 0 & 1 \end{array}\right)$, where T∈SE(3). R∈SO(3) and $t\;\in \;R^{3}$ are translational and rotational elements and can be inverted to the logarithmic map using:

(5) $$\begin{array}{c} \delta \;=\;\left(\begin{array}{c} p\\ \omega \end{array}\right)\;=\;\left(\begin{array}{c} x\\ y\\ \begin{array}{c} z\\ \omega_{1}\\ \omega_{2}\\ \omega_{3} \end{array} \end{array}\right),\\ \theta =arccos\left(\frac{tr\left(R\right)-1}{2}\right),\\ ln\left(R\right)=\frac{\theta }{\sin\theta }\cdot \left(R-R^{T}\right), \end{array}$$

where θ is the axis angle calculated from Equation (5). ω can be recovered from the off-diagonal elements of $ln\left(R\right)$ and $p\;=\;V^{-1}t$. These pose estimates from SE(3) composition layers are referred to as unrelated stacks due to the reason that these are estimated based on the predicted poses of $\left\{T_{t\to t+1},T_{t+1\to t+2},T_{t+2\to t+3}\right\}$ corresponding to image stacks $\left\{I_{t,t+1},I_{t+1,t+2},I_{t+2,t+3}\right\}$ in the forward pass from:

(6) $$\begin{array}{c} T_{t\to t+2}=T_{t\to t+1}⊙T_{t+1\to t+2},\\ T_{t\to t+3}=T_{t\to t+1}⊙T_{t+1\to t+2}⊙T_{t+2\to t+3},\\ T_{t+1\to t+3}=T_{t+1\to t+2}⊙T_{t+2\to t+3}, \end{array}$$

where ⊙ represents the dot product.

### 2.5. Loss Function

The training process consists of adjusting the network parameters θ by minimizing the deviation between predicted $\hat{u_{t}}$ and ground truth $u_{t}$ poses. The conditional probability of the VO problem can be formulated, and optimal parameters $\theta^{*}$ can be estimated by maximizing the following objective:

(7) $$\begin{array}{c} P\left(U_{t}|I_{t}\right)\;=\;P\left(u_{1},\;u_{2},\;u_{3},\;\ldots \ldots ,\;u_{t}|i_{1},\;i_{2},\;i_{3},\ldots \ldots .,\;i_{t}\right),\\ \theta^{*}=\underset{\theta }{\mathrm{argmax}}P\left(u_{t}|I_{t},I_{t+1};\theta \right). \end{array}$$

This method uses a homoscedastic uncertainty-based loss function to automatically choose the weighting coefficient between translational and rotational counterparts. The selected homoscedastic loss function consists of two uncertainty quantification regularization terms $\left({\hat{s}}_{p},\;{\hat{s}}_{\omega }\right)$ as given in Equation (8):

(8) $$Loss=\frac{1}{t}\sum_{k=1}^{t}L_{p}\exp\left(-{\hat{s}}_{p}\right)+{\hat{s}}_{p}+L_{\omega }\exp\left(-{\hat{s}}_{\omega }\right)+{\hat{s}}_{\omega },$$

where $L_{p}={∥\hat{p_{t}}-p_{t}∥}_{2}^{2}$ and $L_{\omega }={∥\hat{\omega_{t}}-\omega_{t}∥}_{2}^{2}$ are the euclidean distance between ground truth $\left(p_{t},\;\omega_{t}\right)$ and predicted $\left(\hat{p_{t}},\;\hat{\omega_{t}}\right)$ translational and rotational elements, respectively. Standard networks solely minimize the relative transformational errors. Optimizing the nearest frames by enforcing the geometric constraints using composite poses jointly is the key to maintain lesser drifts. The total loss term consists of directly estimated relative poses with estimated composite poses are written as Equation (9):

(9) $$\begin{array}{c} {Loss}_{relative}={Loss}_{t\to t+1}\;+\;{Loss}_{t+1\to t+2}\;+\;{Loss}_{t+2\to t+3},\\ {Loss}_{composite}={Loss}_{t\to t+2}\;+\;{Loss}_{t\to t+3}\;+\;{Loss}_{t+1\to t+3},\\ {Loss}_{total}={Loss}_{t\to t+1}\;+\;{Loss}_{t+1\to t+2}\;+\;{Loss}_{t+2\to t+3}\;+\;{Loss}_{t\to t+2}\;+\;{Loss}_{t\to t+3}\;+\;{Loss}_{t+1\to t+3},\\ {Loss}_{total\;(DA)}={Loss}_{t\to t+j}\;+\;{Loss}_{t+j\to t+k}\;+\;{Loss}_{t+k\to t+l}\;+\;{Loss}_{t\to t+k}\;+\;{Loss}_{t\to t+l}\;+\;{Loss}_{t+k\to t+j}, \end{array}$$

where ${Loss}_{total\;(DA)}$ is the loss function for samples with data augmentation (*DA*), and *j*, *k*, *l* are the random values ranging from 0 to 4.

## 3. Experiments

This section presents the details of the performance evaluation of the proposed method. First, the software and hardware environment used to train and test the proposed method with a set of selected hyperparameters are presented. Second, details of the benchmark and evaluation metrics associated are described. Next, the importance of DA in the VO task is presented by choosing the varying amount of augmented data. Performance of the related works is compared relatively to current method to evaluate the efficiency and accuracy of the current windowed deep optimization technique. Finally, a detailed ablation study is performed on the network to visualize the importance of windowed optimization with a detailed run-time analysis.

### 3.1. Implementation Details

The network was trained and tested using PyTorch framework in Python on Nvidia 2080S GPU with a memory of 8 GB and Intel i9-10900F at 2.80 GHz. An Adam optimizer with default setting of $\beta_{1}=0.9,\beta_{2}=0.999$ was used, as presented in Reference [34]. The initial learning rate of 0.001 with a half decay rate for every 30 epochs until 150 epochs was selected to train the network. Even though our model only consumes one-fourth of the total GPU available, batch size remained at 32 for training and testing.

### 3.2. Dataset

We used the KITTI VO benchmark [21] to train and test WPO-Net. The dataset consists of 21 sequences composed of 23,201 images; 11 of the 21 sequences are available with ground truth pose estimates. For this work, we adopted a split used in References [31,32,33,35,36,37], which reserves 00-08 sequences for training and 09, 10 sequences for testing. A station wagon is used to collect the dataset in outdoor environments with a frequency of 10 frames per second and compromises of challenging scenarios with dynamic objects. The default image size of the images in the dataset is 1241 × 376, and the images are resized to half for training and testing the proposed network to constrain the computational cost. Training data is augmented using a temporal skipping technique, and no DA is involved while testing the network.

Three evaluation metrics, namely absolute trajectory error (ATE(m)), translational error $\left(t_{rel}\left(\%\right)\right)$, and rotational error $(r_{rel}$(deg/100 m)), are used to efficiently evaluate within various sizes of samples of the present method and related works. Translational and rotational errors are obtained by averaging the subsequence errors from 100 to 800 m with an interval of 100 m.

### 3.3. Effects of Data Augmentation

Data is one of the crucial components for any learning-based paradigm, such as deep learning. This section emphasis on a long-standing yet challenging problem in training deep networks. The majority of supervised learning works adapted a manual weighting approach to tune the balance between the rotational and translational elements, which is time-consuming and needs an extensive parameter search space. However, it is very difficult to derive a quantitative measure between rotational and translational samples in the VO task, and, to avoid these data-related uncertainties and to adaptively weight the elements, a homoscedastic based loss is used. Another interesting direction is to increase the size of the available dataset with techniques, such as random sampling, cropping, and noise addition. A temporal skipping technique is used for this study to augment the data, and the effects of different percentages of augmentation with respect to evaluation metrics are shown in Table 2.

**Figure 2:**
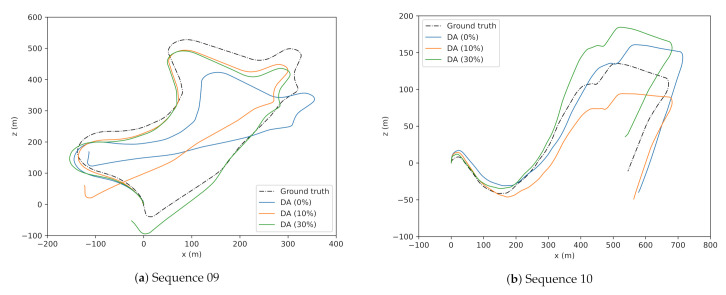
Trajectories of sequences 09 (**a**) and 10 (**b**) under different data augmentation (DA) quantities. *X* and *Y*-axis represent motion along the *Z* (forward) and *X* (left/right) axis of the vehicle in the vehicular frame.

The predicted trajectories of the best model DA (30%), second-best DA (10%) are plotted against the ground truth in Figure 2. The overall estimated trajectory trained with DA 30 percent performed well on ATE and translational error $\left(t_{rel}\right)$. This study considers ATE as one of the significant evaluation metrics in the aspects of VO tasks to reduce the drift and is often underemphasized.

**Figure 3:**
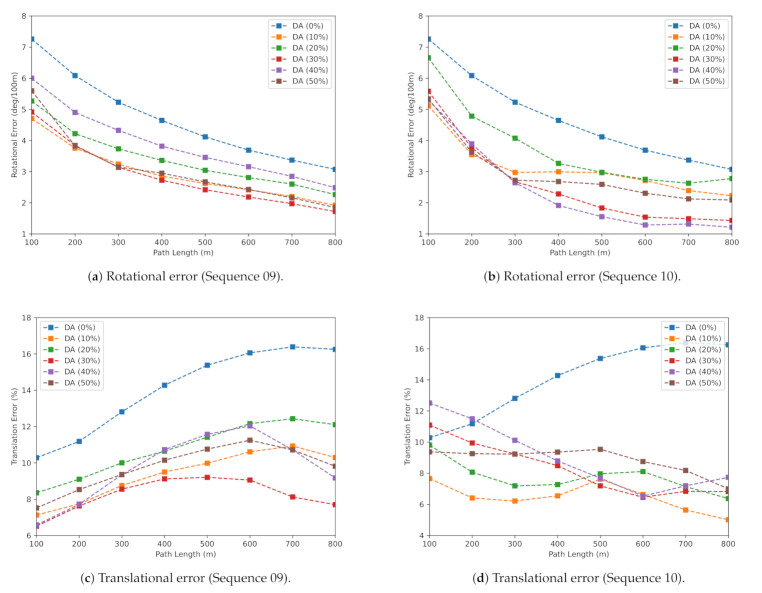
Comparison of rotational and translational errors of different DA quantities at subsamples of varying length (100 m, 200 m, 300 m, …, 800 m) sequences 09 and 10.

From the experiments, it is evident that increasing the dataset by augmenting does not always result in higher accuracies, especially in a complex multi-view geometry problem, such as VO. The best model for comparison with other related works is chosen to be the dataset with DA (30%). Though the dataset with DA (10%) performed superior to other splits in terms of translational error, the dataset with DA (30%) outperformed it over the other two evaluation metrics. Rotational and translation errors of models trained on different augmentation split and tested on sequences 09, 10 for subsamples are shown in Figure 3. From Figure 3c,d, it can be observed that the model trained on DA with 30 percent is stable and accurate compared to other splits. Similarly, from Figure 3a,b, DA (30%) performed superior to other splits. Though DA (30%) is lagging behind DA (10%) in a singular case (translational error $\left(t_{rel}\right)$), overall performance of DA (30%) is better compared to others, and this model is used to compare with the related works in the next section.

### 3.4. Comparison with Related Works

This section evaluates the proposed method with other significant published works. The proposed WPO-Net is evaluated across three different algorithms. First, Monocular VISO2 [18] and ORB-SLAM [6] are used to evaluate against the state of art algorithms. Second, a supervised version of Reference [35], DeepVO [11], and Flowdometry [12] are employed to compare with the supervised learning-based methods. Though DeepVO and Flowdometry are some of the most prominent supervised learning-based methods, different splits were used for training and testing. To effectively deal with such train-test split discrepancies in comparison with other methods, the average translation, and rotational errors across all sequences are used. Finally, unsupervised learning-based methods, such as in References [31,32,33,36,37], are included in the comparison with WPO-Net in Table 3.

Although the performance of WPO-Net is slightly unsatisfactory on sequence 09 against VISO2M, the overall performance advantage is higher and accurate. In addition, the current method avoids the complex pipeline involving numerous subsystems, such as VISO2M and ORB-SLAM. On the other hand, WPO-Net performed significantly better on sequence 09 than any other learning-based methods used for comparison. Supervised learning-based methods take the advantage of implicitly learning the scale during the training process. The overall rotational error is minimal in comparison with other methods. This experiment verifies the ability of the learning-based windowed pose optimization technique in improving the accuracy of the system.

### 3.5. Ablation Study

**Figure 4:**
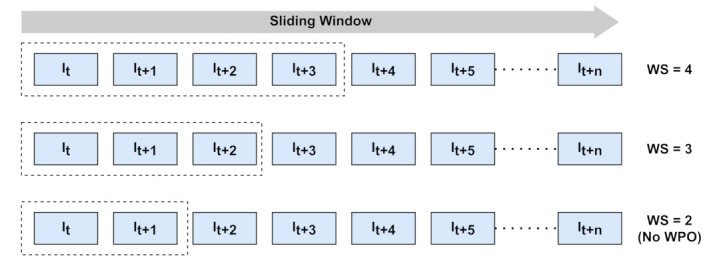
An illustration of the number of images taken as input to the network for WS=2,3,4.

This section includes the experimentation on the proposed WPO-Net to examine the efficiency of learning-based windowed pose optimization. The conclusion is drawn by training and testing the network with three different window sizes (WS). The WS defines the number of consecutive images used for every single backpropagation. Let WS be equal to *n* images, and the number of times the network is forward propagated is given by (n−1) with a single backpropagation. When WS=2, the network by default acts as a standard supervised network with one sample input and one sample output. The three different window sizes are selected to observe the efficiency of windowed pose optimization by examining the evaluation metrics. Figure 4 illustrates the number of images used for a single iteration as the windows slide towards the right.

All the networks used for comparison in this section are trained and tested with the same split, as mentioned in Section 3.3, with 30 percent of DA. The network with WS=4 was the one used to compare with related work, and the data is derived from Section 3.4. The results of the evaluation metrics of different WS’s are presented in Table 4.

**Figure 5:**
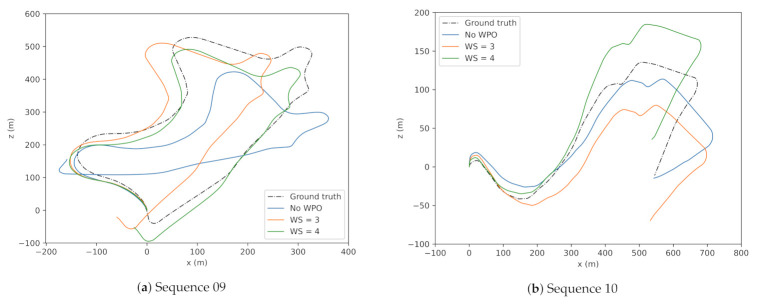
Trajectories of sequences 09 (**a**) and 10 (**b**) under different window sizes (WS). *X* and *Y*-axis represent motion along the *Z* (forward) and *X* (left/right) axis of the vehicle in the vehicular frame.

This experiment provides clear evidence of increased performance while using windowed optimization. This technique also can be viewed as a resemblance to windowed bundle optimization used in state-of-the-art VO methods. It is also important to consider the computational overheads during training with a larger *WS*. Thus, to limit the total training time of WPO-Net, WS is limited to 4. Furthermore, the predicted trajectories of WS=2,3,4 are illustrated in Figure 5.

Time taken for inference and training are measured by using a batch size of 2 averaged over hundred iterations. The inference, training time on GPU is 3.98, 19.54 and CPU is 7.87, 41.32 ms, respectively. The total parameter count of WPO-Net is 0.48 million, which makes it a light and affordable network to run on embedded controllers. Comparison of run-time analysis of WPO-Net with other methods is not included because the hardware used is different from method-to-method.

## 4. Conclusions

In this paper, an optimization method for learning-based VO is proposed. The proposed method can reduce overall trajectory drift and improves the accuracy of the system. From experiments, it was clear that increasing the data augmentation over a specific point degrades the performance. The proposed method outperformed most of the unsupervised methods included in comparison on the KITTI dataset. This method achieved the least rotational error than any other methods included in the comparison. The mean rotational error was improved by 13.06% compared to Reference [36], which is the best among the related works used to compare. It is certainly helpful to also note that learning-based methods included in the evaluation consist of a larger number of parameters than WPO-Net. The inference time of the proposed method on the CPU is 7.87 ms. In future work, we will validate the real-time performance of the proposed WPO-Net, along with some generalization tests.

## Figures and Tables

**Table 1:** Architecture of the feature encoder. The filter’s size decreases as the depth of the network increases.

Layer	Kernel Size	Channels	Stride	Dilation
Input	-	2	-	-
Layer-1	3 × 9	16	2	2
Layer-2	3 × 9	16	2	1
Layer-3	3 × 7	32	2	2
Layer-4	3 × 7	32	2	1
Layer-5	3 × 5	64	1	2
Layer-6	3 × 5	64	1	1
Layer-7	2 × 2	64	2	1

**Table 2:** Effects of varying quantities of DA on MPO-Net (the least error results are high-lighted in bold text).

DA (%)	ATE (m)	Trans $t_{\mathrm{rel}}$ (%)	Rot $r_{\mathrm{rel}}$ (deg/100 m)
0	91.82	12.82	5.07
10	57.52	**7.95**	3.27
20	96.81	9.31	3.91
30	**48.76**	8.57	**3.06**
40	94.03	9.70	3.49
50	79.06	9.38	3.28

**Table 3:** Comparative results on the KITTI benchmark (data is extracted from the corresponding works/citations, the least error results are high-lighted in bold text).

Method	Sequence 09		Sequence 10		Avg	
	Trans $t_{\mathrm{rel}}\left(\%\right)$	Rot $r_{\mathrm{rel}}$ (deg/100 m)	Trans $t_{\mathrm{rel}}\left(\%\right)$	Rot $r_{\mathrm{rel}}$ (deg/100 m)	Trans $t_{\mathrm{rel}}\left(\%\right)$	Rot $r_{\mathrm{rel}}$ (deg/100 m)
VISO2M [18]	**7.08**	**1.15**	41.60	32.99	24.34	17.07
ORB-SLAM [6]	-	-	86.51	98.90	30.01	35.53
Flowdometry [12]	12.64	8.04	11.65	7.28	11.42	6.92
DeepVO [11]	-	-	8.11	8.83	**5.96**	6.12
SfM Learner [31]	17.84	6.78	37.91	17.78	27.88	12.28
GeoNet [32]	43.76	16.00	35.6	13.80	39.68	14.90
Zhan et al. [36]	11.92	3.60	12.62	3.43	12.27	3.52
Wang et al. [35]	9.30	3.50	**7.21**	3.90	8.26	3.70
SC-SfM [37]	11.20	3.35	10.10	4.96	10.65	4.16
CM-VO [33]	9.69	3.37	10.01	4.87	9.85	4.12
WPO-Net (proposed)	8.19	3.02	8.95	**3.12**	8.57	**3.06**

**Table 4:** Effects of different window sizes (ws) on MPO-Net (the least error results are high-lighted in bold text).

WS (n)	Forward Passes (n−1)	ATE (m)	Trans $t_{\mathrm{rel}}\left(\%\right)$	Rot $r_{\mathrm{rel}}$ (deg/100 m)
2 (no WPO)	1	98.30	12.95	4.79
3	2	84.25	9.41	3.48
**4**	**3 **	**48.76**	**8.57**	**3.06**

## Data Availability

The KITTI dataset [21] used for this study is openly available at http://www.cvlibs.net/datasets/kitti/ (accessed on 15 June 2021).
